# Imposing assertions in Maude via program transformation

**DOI:** 10.1016/j.mex.2019.10.035

**Published:** 2019-11-06

**Authors:** María Alpuente, Demis Ballis, Julia Sapiña

**Affiliations:** a VRAIN (Valencian Research Institute for Artificial Intelligence), Universitat Politècnica de València, Camino de Vera s/n, Apdo 22012, 46071 Valencia, Spain; b DMIF, University of Udine, Via delle Scienze, 206, 33100 Udine, Italy

**Keywords:** Transformation method for enforcing system invariants in Maude programs, Assertion enforcement, Automated program transformation, Program repair, Equational rewriting, Rewriting logic, Maude

## Abstract

Program transformation is widely used for producing correct mutations of a given program so as to satisfy the user’s intent that can be expressed by means of some sort of specification (e.g. logical assertions, functional specifications, reference implementations, summaries, examples). This paper describes an automated correction methodology for Maude programs that is based on program transformation and can be used to enforce a safety policy, given by a set **A** of system assertions, in a Maude program **R** that might disprove some of the assertions. The outcome of the technique is a safe program refinement **R'** of **R** in which every computation is a good run, i.e., it satisfies the assertions in **A**. Furthermore, the transformation ensures that no good run of **R** is removed from **R**'. Advantages of this correction methodology can be summarized as follows.

• A fully automatic program transformation featuring both program diagnosis and repair that preserves all executability requirements.
• A simple logical notation to declaratively express invariant properties and other safety constraints through assertions.
• No dynamic information is required to infer program fixes: the methodology is static and does not need to collect any error symptom at runtime.

## Graphical abstract

**Figure:**
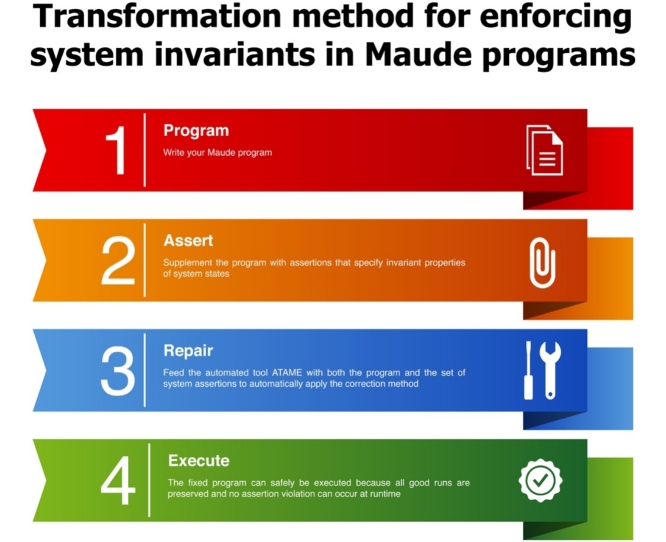

**Specification Table**

**Table:** 

Subject Area:	*Computer science*
More specific subject area:	*Methods and tools to guarantee software quality and trustworthiness*
Method name:	*Transformation method for enforcing system invariants in Maude programs*
Name and reference of original method:	S*tatic Correction Method for Maude Programs with Assertions* *M. Alpuente, D. Ballis, and J. Sapiña, Static Correction of Maude Programs with Assertions. Journal of Systems and Software vol. 153, pages 64-85, July 2019.*
Resource availability:	http://safe-tools.dsic.upv.es/atame/

## Method details

### Short introduction regarding the method applicability and motivation

This paper describes an automated correction methodology that can be applied to impose safety properties on concurrent and nondeterministic software systems that are modelled as Maude programs. Nonetheless, the core idea of our correction transformation can be transferred to virtually any rewriting-based programming language, from simple term rewriting systems and rule-based languages such as CafeOBJ, OBJ, ASF + SDF, and ELAN, to widespread functional languages such as Haskell and Erlang, provided that the transformation preserves the executability conditions required by the language. Indeed, the proposed correction method transforms program rules into guarded program rules whose conditions supersede the (external) safety assertion checks and are simply evaluated by using the very same rewriting infrastructure of the language. Therefore, the provided assertion checking mechanism can be embedded into any setting that supports rewriting with an effort that depends on the complexity of the chosen formal framework.

**Fig. 1:**
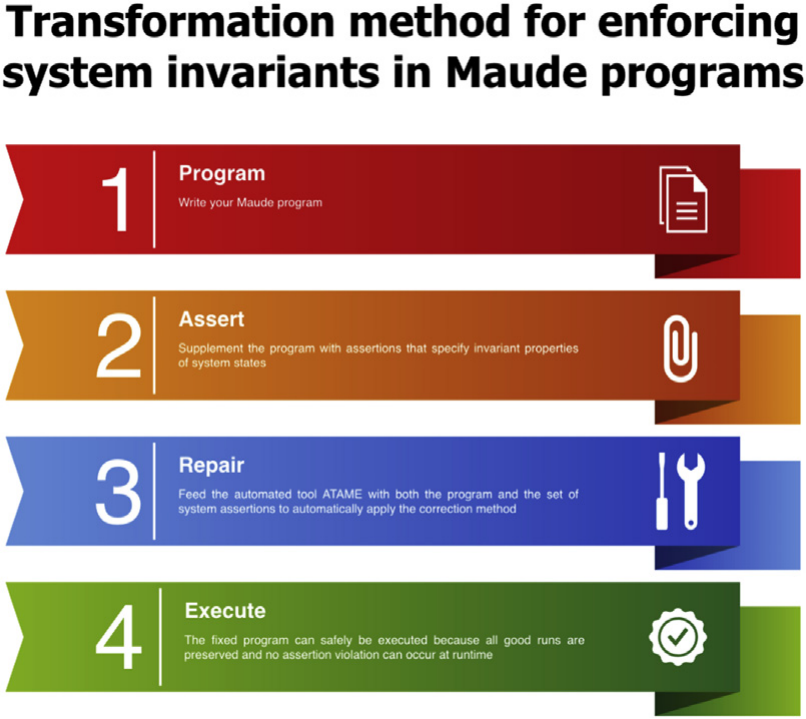
Basic correction procedure.

In the following, we outline the correction procedure for repairing Maude programs with respect to a safety policy that is expressed as a set of system assertions; a similar modus operandi can be followed to replicate this method in different rewriting frameworks such as those mentioned above. The advantage of the technique is that more refined versions of a program can be incrementally built without any programming effort by simply adding new safety constraints into the set of assertions. This makes it possible to adapt existing Maude programs to predefined safety policies and allows the inexperienced user to largely forget about Maude syntax and semantics. An infographic that outlines the basic steps of the correction methodology is given in Fig. 1.

### On the rewrite framework

Maude [2] is a high-performance language and system that efficiently implements Rewriting Logic [4], which is a logic of change that seamlessly unifies a wide variety of models of concurrency. A Maude program **R** is essentially made up of two components, E and R, where

• E is a canonical (membership) equational theory that models system states as terms of an algebraic data type, and
• R is a set of rewrite rules that define transitions between states and which is assumed to be coherent w.r.t. the equations in the set E.

Canonicity of E and coherence between R and E are fundamental executability properties that guarantee the soundness and completeness of Maude’s evaluation mechanism [3].

Algebraic structures often involve axioms like associativity, commutativity, and/or identity (also known as unity) of function symbols, which cannot be handled by ordinary term rewriting but instead are handled implicitly by working with congruence classes of terms. More precisely, the membership equational theory E is decomposed into a disjoint union E = D ∪ Ax, where

• the set D consists of (conditional) membership axioms (i.e., axioms that assert the type of some terms) and equations that are implicitly oriented from left to right as rewrite rules (and operationally used as simplification rules), and
• Ax is a set of algebraic axioms that are implicitly expressed as function attributes and are mainly used for Ax-matching.

The system evolves by rewriting states using equational rewriting, i.e., rewriting with the rewrite rules in R modulo the equations and axioms in E. For the sake of simplicity, we only consider *topmost* Maude programs, that is, Maude programs in which rewrites can only happen at the state top level position. This implies that no local state changes are allowed: in other words, each rewrite step completely replace a state s_1_ with a new term representing the derived state s_2_. In [1], increasingly involved Maude program structures are considered (such as *topmost modulo* Ax rewrite theories and Russian doll theories that support system states with recursively nested structures).

In our framework, system safety properties are specified by means of assertions, that is, logical statements of the form S | ϕ, where S is a term (the *state template*) and ϕ is a quantifier-free, first-order logic formula (the *state invariant*). An assertion S | ϕ holds in a system state s iff, for every subterm of s that matches (modulo E) the algebraic structure of the state template S with substitution σ, the constraints given by the instantiated formula φσ are satisfied. In our scenario, the notion of satisfaction of a (closed) instance φσ of φ boils down to reducing φσ to its truth value via equational rewriting. If an assertion does not hold in a system state s, we say that there is an assertion violation in s.

Maude's formal tools are numerous and perform different analysis and verification tasks, either statically (e.g., Maude's theorem prover and model checker) or dynamically (Maude's assertion checker); see [1] for references. However, to the best of our knowledge, there is no previous methodology for automated safety enforcement in Maude.

### The proposed method

Our correction method is based on a two-phase program transformation technique that allows a Maude program **R** to be refined into a program **R**' w.r.t. a set of assertions **A** as follows. Let us assume that the program **R** consists of the equation set E and the rewrite rule set R.

1 The first phase translates the assertion set **A** into an executable equational definition Eq(**A**) that can be used to detect assertion violations within system states. Roughly speaking, given a system state s, a violation of some assertion in **A** is detected in s whenever a renamed apart version s’ of s can be simplified into the special constant fail by using the equational theory E of **R** extended with Eq(**A**).

Specifically, each assertion (S | ϕ) is encoded by a conditional equation in Eq(**A**) of the form

**Figure:**
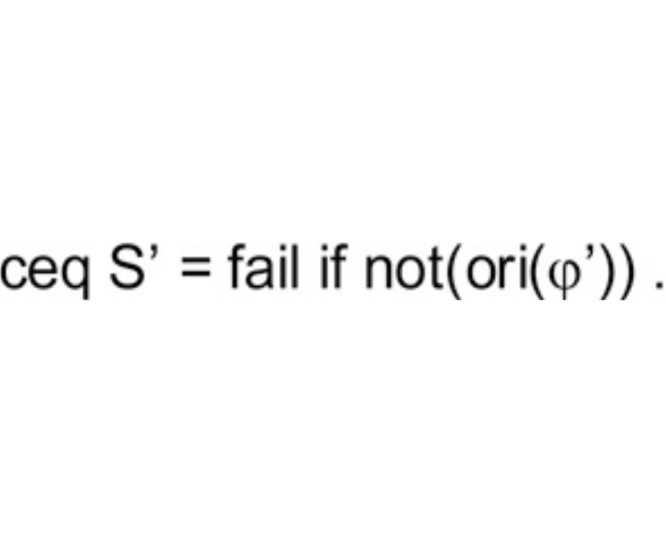

such that

• S’ is a renamed apart version of the state template S where each operator f in S has been replaced by a new operator f’^3^ ;
• fail is a fresh new constant that does not occur in R;
• ori(t’) is a function that takes a renamed apart term t’ and restores its original version t, that is, ori(t’) = t.

Note that assertion checking is executed over renamed versions of the original program states, while logic formulas are evaluated by using the original operators of **R**. Renaming is key to neatly separate assertion checking from system computations and avoid interferences that might jeopardize termination, confluence and/or coherence properties in the repaired program (for a detailed discussion on renaming, see [1]).

2 The second phase transforms the original rewrite rules of **R** into guarded, conditional rewrite rules that can only be fired if no system assertion is violated. Intuitively, this is achieved by transforming each rewrite rule r : (λ → ρ if C) of **R** into a refined version r’: (λ → ρ if C ∧ ren(ρ) =/= fail) of r, which contains the extra constraint ren(ρ) =/= fail that holds when the renamed apart instances of the right-hand side ρ of the rule r cannot be simplified to fail by using the extended equational theory E ∪ Eq(**A**).

This way, we ensure that any state transition from state s_1_ to state s_2_ is enabled in the program **R’** only if s_2_ is a safe state, that is, every assertion of **A** holds in s_2_.

As an important advantage of the method, executability conditions of R and E are preserved by the correction transformation. Furthermore, the methodology copes with infinite space states and does not require the knowledge of any failing run. A rigorous and complete formalization of the method can be found in [1].

### A typical correction transformation session

**Fig. 2:**
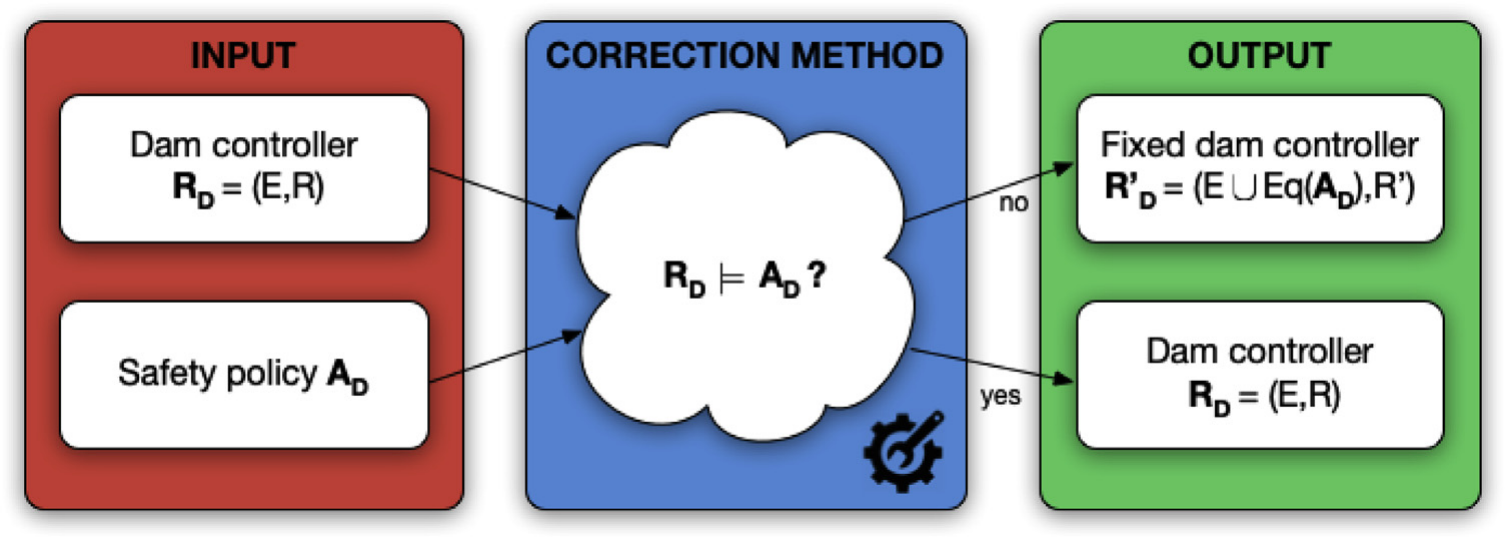
Correction workflow.

To show how our correction methodology works in practice, we consider a topmost Maude program **R_D_** that specifies a simple dam controller for monitoring and managing the water volume of a basin. The workflow of the correction methodology is depicted in Fig. 2.

In the sequel, variable names are fully capitalized. We assume that the dam model is provided with a spillway called s which has three possible aperture widths of increasing discharge capacity c, o1, o2. A spillway configuration is formally specified by a term [s,O], where O belongs to the set {c,o1,o2}. System states are defined by terms of the form

**Figure:**
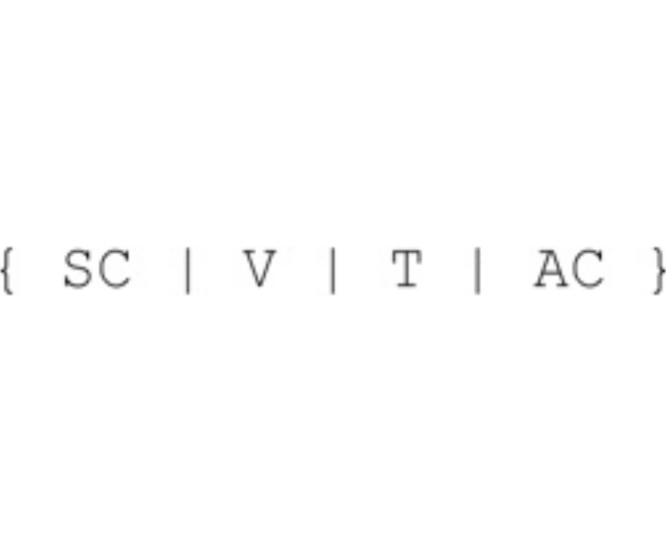

where SC is a spillway configuration, V is a rational number that indicates the basin water volume (in m^3^), T is a natural number that timestamps the current configuration, and AC (*aperture command)* is a Boolean flag that enables changes of the spillway aperture widths only when its value is true.

To keep the exposition simple, we assume that the basin water inflow is constant, while the basin outflow depends on the aperture width of the current spillway configuration. Basin inflow and outflow are measured in m^3^/min and are specified by the following Maude equations

**Figure:**
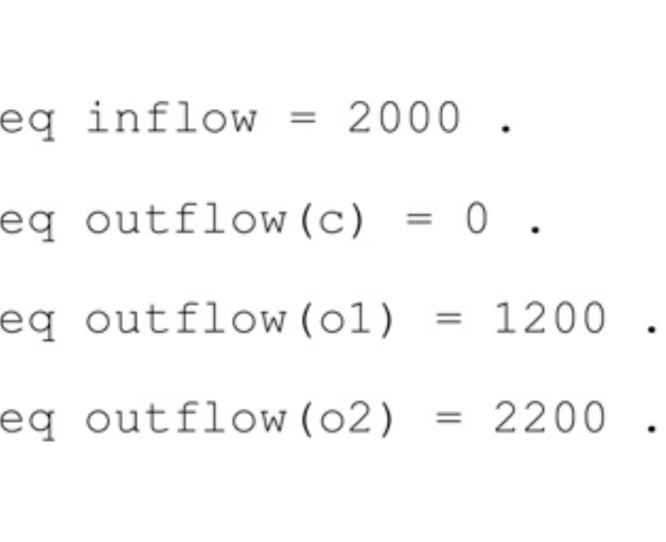

Note that more realistic scenarios could be easily defined by specifying more sophisticated basin inflow and outflow functions.

The dam controller dynamics is modeled by the following eight rewrite rules, which implement system state transitions.

**Figure:**
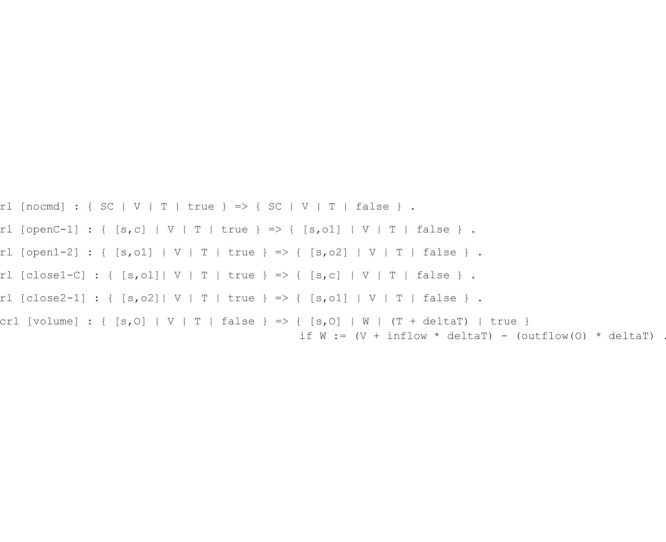

The openX-Y rewrite rules progressively increment the aperture width of the spillway s (e.g., the rule open1-2 increases the aperture of the spillway s from level open1 to level open2). Dually, closeX-Y rewrite rules progressively decrease the aperture width of a spillway. The rule nocmd specifies the empty command, which basically states that no action is taken on the spillway configuration by the dam controller at time instant T. The rule is fired only when the AC flag is enabled, and its application disables the flag to allow a new basin water volume to be computed in the next time instant. These rules implement instantaneous spillway modifications that do not change the time instant or the basin water volume.

The temporal evolution of the basin water volume is specified by the conditional rewrite rule volume that computes the volume W at time T + deltaT, given the input volume V at time T. The parameter deltaT is measured in minutes and can be set by the user. The volume computation changes the input volume V by adding the water inflow and subtracting the corresponding water outflow over the deltaT interval.

The use of the AC flag in the rule definitions guarantees a fair interleaving between the applications of the rule volume and the remaining rewrite rules. Specifically, this implies that a new basin water volume is computed after each spillway aperture width modification.

Note that computations in **R_D_** may reach potentially hazardous system states (e.g., an extremely high water volume), since **R_D_** does not implement any spillway management policy that safely restricts the applications of the rewrite rules. Thus, the following companion assertion set **A_D_** to be enforced is specified in order to apply our correction transformation:

**Figure:**
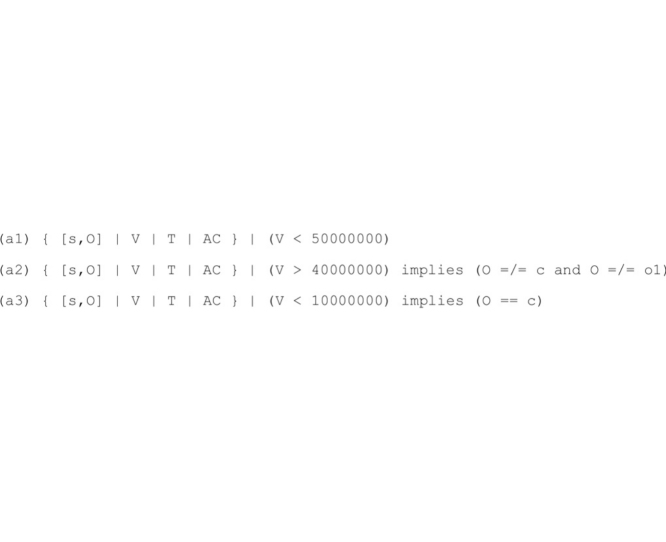

Roughly speaking, assertion a1 states that, in every system state, the basin water volume must be less than 50 million m^3^ to avoid dam bursts and potentially disastrous floods. Assertion a2 specifies that, whenever the basin water volume is greater than 40 million m^3^, the spillway must be fully open (i.e., aperture width o2). Assertion a3 requires the complete closure of the spillway when the basin water volume is particularly low (10 million m^3^).

The *first phase* of our correction method generates the equational theory Eq(**A_D_**) that includes the following encodings of the assertions in **A_D._**

**Figure:**
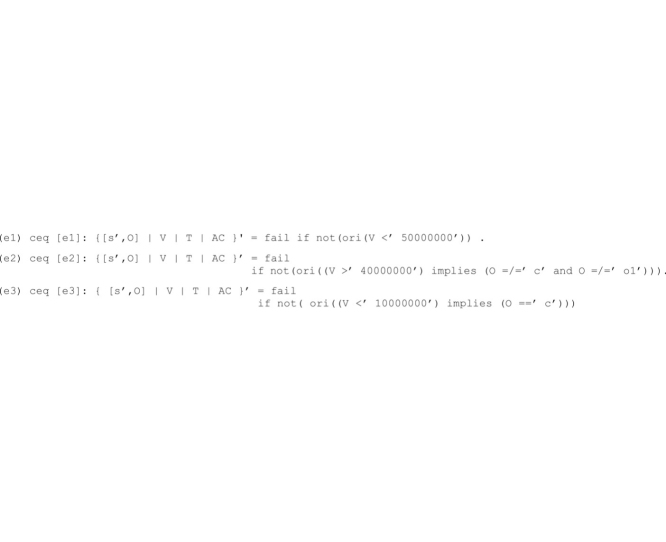

These equations allow any renamed system state to be rewritten to fail whenever the corresponding assertion is violated.

The *second phase* transforms each rewrite rule of **R_D_** into their refined conditional counterpart as follows:

**Figure:**
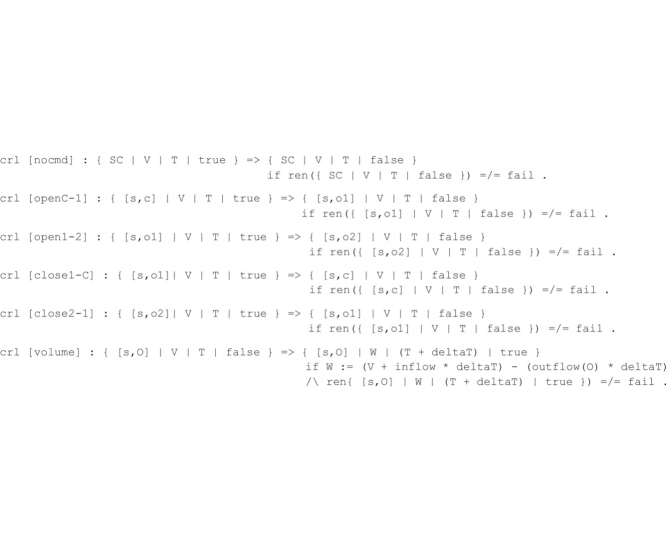

By using the refined rules above, any state transition from a state s_1_ to a state s_2_ occurs only when s_2_ does not violate the assertions in **A_D,_** thereby enforcing a safe behavior of the corrected dam controller.

### Method implementation and validation

The correction methodology has been implemented in the ATAME system that is available at http://safe-tools.dsic.upv.es/atame. We conducted a thorough experimental evaluation using ATAME that demonstrates good performance (regarding code size, execution time, and program transformation time) for a number of benchmarks that are available and fully described within the ATAME web platform and in [1]. As shown in [1], transformation times are almost negligible, and moreover, running the corrected program **R**' in Maude is more than 50% faster on average than running the original program **R** in a monitored environment that implements runtime assertion checking.

**Fig. 3:**
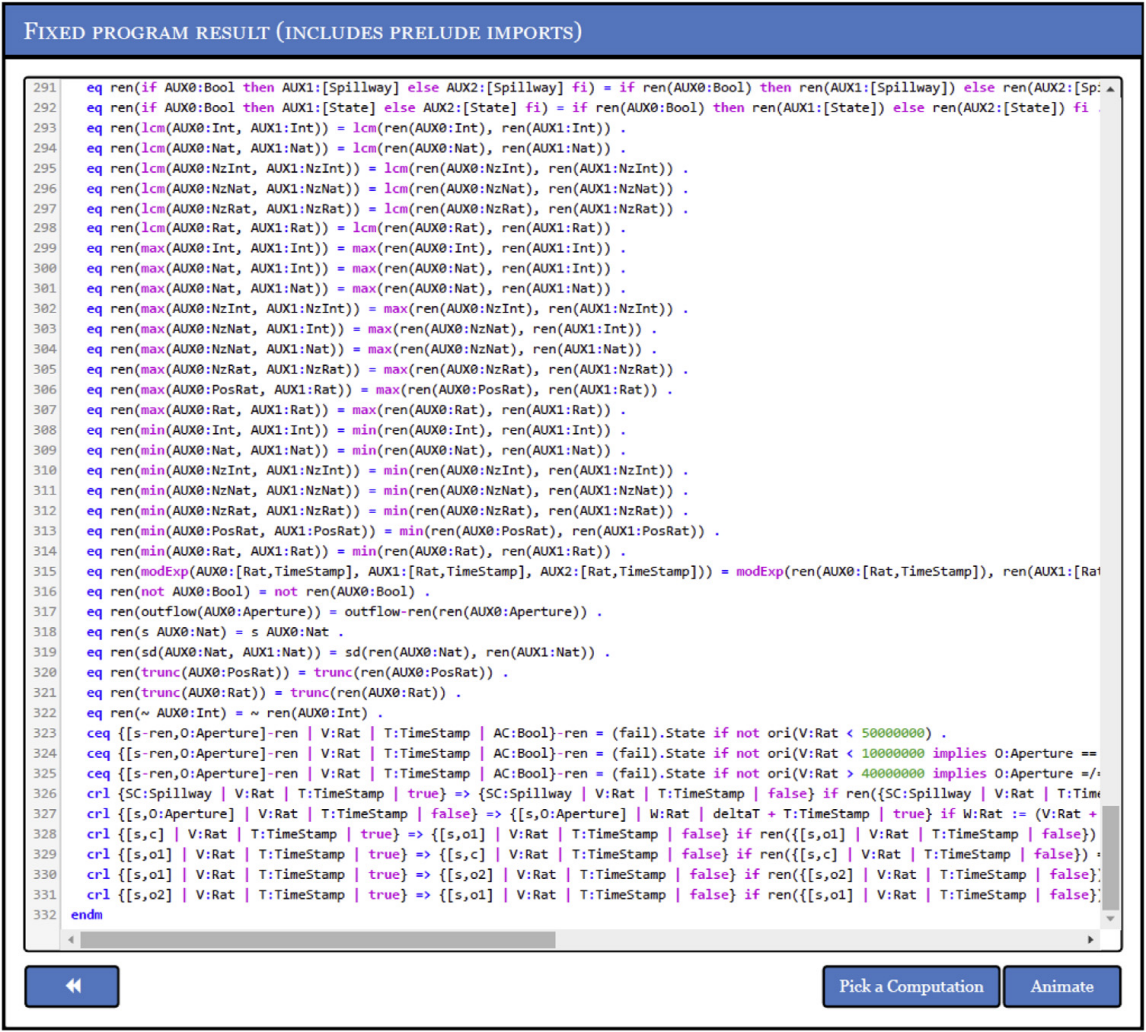
Fixed dam controller **R’_D_**.

Maude programs can be either uploaded to ATAME as simple “.maude” files or written from scratch. Once the intended assertions have been also introduced inside a dedicated edit box, the correction procedure can be executed by simply clicking the “Fix Program” button, which delivers a coerced version of the program whose computations respect all the imposed assertions. Fig. 3 shows a fragment of the dam controller **R’_D_** that has been automatically fixed by ATAME.

## Funding

This work has been partially supported by the EU (FEDER) and the Spanish MINECO under grant RTI2018-094403-B-C32, and by Generalitat Valenciana under grant PROMETEO/2019/098.

## Declaration of Competing Interest

The Authors confirm that there are no conflicts of interest.

## Footnotes

3 Note that, in the case of mixfix operators, we just rename one operator symbol. For instance, the binary, mixfix operator < _ | _ > would be renamed < _ | _ >’.
